# A developmental assessment of clinical reasoning in preclinical medical education

**DOI:** 10.1080/10872981.2019.1591257

**Published:** 2019-04-01

**Authors:** Alice A. Min Simpkins, Bryna Koch, Karen Spear-Ellinwood, Paul St. John

**Affiliations:** a Department of Emergency Medicine and the Assistant Dean for Faculty Development in the College of Medicine – Tucson, University of Arizona, Tucson, AZ, USA; b Arizona Center for Rural Health, University of Arizona College of Public Health, Tucson, AZ, USA; c Department of Obstetrics and Gynecology and the Director of Faculty Instructional Development at the College of Medicine - Tucson, University of Arizona, Tucson, AZ, USA; d Department of Cellular and Molecular Medicine at the College of Medicine-Tucson, University of Arizona, Tucson, AZ, USA

**Keywords:** Clinical reasoning, medical education, assessment

## Abstract

**Background**: Clinical reasoning is an essential skill to be learned during medical education. A developmental framework for the assessment and measurement of this skill has not yet been described in the literature.

**Objective**: The authors describe the creation and pilot implementation of a rubric designed to assess the development of clinical reasoning skills in pre-clinical medical education.

**Design**: The multi-disciplinary course team used Backwards Design to develop course goals, objectives, and assessment for a new Clinical Reasoning Course. The team focused on behaviors that students were expected to demonstrate, identifying each as a ‘desired result’ element and aligning these with three levels of performance: emerging, acquiring, and mastering.

**Results**: The first draft of the rubric was reviewed and piloted by faculty using sample student entries; this provided feedback on ease of use and appropriateness. After the first semester, the course team evaluated whether the rubric distinguished between different levels of student performance in each competency. A systematic approach based on descriptive analysis of mid- and end of semester assessments of student performance revealed that from mid- to end-of-semester, over half the students received higher competency scores at semester end.

**Conclusion**: The assessment rubric allowed students in the early stages of clinical reasoning development to understand their trajectory and provided faculty a framework from which to give meaningful feedback. The multi-disciplinary background of the course team supported a systematic and robust course and assessment design process. The authors strongly encourage other colleges to support the use of collaborative and multi-disciplinary course teams.

## Introduction

Clinical reasoning is a necessary skill for physicians, and teaching this skill is an important aspect of medical education. Integrating the instruction of clinical reasoning and assessment of this skill has proven to be challenging and variable, particularly within the pre-clinical phase of undergraduate medical education. While several strategies to assess clinical reasoning of learners have been utilized, a developmental approach to measuring and assessing the progression of these skills has not yet been described in medical education literature.

There is no single ‘gold standard’ for assessing clinical reasoning. Two approaches, patient management problems and computer-based exam projects, ask students to indicate what clinical examinations and diagnostic tests they would perform to narrow the differential diagnosis [1]. These approaches revealed challenges and errors, including inter-rater reliability, variability in the predictability of performance scores in terms of tracking long-term development of clinical reasoning, and the observation that experienced physicians did not perform as well as medical students on some of the same cases or decision-making items [1]. Other strategies shared in the literature include script concordance and Think Aloud protocols [2–4]. The script concordance test is based on the understanding that experienced clinicians have sets of knowledge or scripts, that are used to understand situations and make decisions related to diagnosis and treatment [2]. Learners are provided with a short clinical vignette and asked how an additional piece of new information affects their decisions regarding diagnosis, investigational study, or therapy [2,3]. These responses are then compared to responses by a panel of experts [2,3]. The Think Aloud method calls for individuals to verbalize how they are using the information to generate a solution to a problem [4]. It has been suggested that if learners, particularly in graduate medical education, pause intermittently during clinical presentations to explicitly explain how information is affecting their decision-making, supervisors could assess their clinical reasoning and address errors in real time [4].

Assessing clinical reasoning skills is difficult because it is not one well-defined skill, but a combination of skills that involves understanding the complexity of a problem and the ability to translate and synthesize existing knowledge and skills to new problems. Assessing variation in the development of this skill set poses challenges. For example, some students might arrive at a diagnosis or plan but cannot articulate how they did, while others might arrive at the correct answer and articulate an approach other than one typically taken. Any assessment process, then, should address both a medical student’s current performance of clinical reasoning and their learning progression toward developing this skill set that calls on the simultaneous exercise of medical knowledge, communication and physical examination skills, and critical and reflective thinking. In other words, assessments should be both context-specific and global – allowing for an assessment of performance over time [1]. In discussing future directions in clinical reasoning assessment, Lang et al. described a milestones approach, a developmental assessment process, as a way to capture both diagnostic reasoning (evaluating decision-making points) and therapeutic reasoning that includes ethical and patient-specific information [1].

In 2014, the University of Arizona College of Medicine – Tucson created a Clinical Reasoning course (CRC) that spans the first three semesters of the pre-clerkship curriculum. CRC evolved from Case-Based Instruction (CBI), a case-based activity that was integrated into each preclinical block course and had the primary goal of expanding students’ medical knowledge. CRC was created as a separate, longitudinal, small-group and case-based course that had the primary goal of developing students’ clinical reasoning skills. The change in course goals was paired with the adoption of an online platform called ThinkShare^TM^, where students explained in writing their thinking about the cases before coming to their small-group sessions. Students progressed through each case in a stepwise process, with opportunities to respond to new clinical or patient information disclosed at progressive junctures.

A challenge for the new course was to devise a system to assess student performance. We describe the development and implementation of a rubric designed to assess the development of clinical reasoning skills in pre-clinical medical education. This rubric reflects the developmental process and ‘milestones’ expected as a learner progresses through a clinical reasoning curriculum.

## Materials and methods

The CRC team implemented a Backward Design process to create the new course goals, learning objectives, and student assessments [5]. An important aspect of the CRC course design process was that the team included individuals from multiple disciplines with advanced degrees in the basic sciences, clinical medicine, education and public health and with expertise in instructional design, assessment, and course/curriculum evaluation. The course goals were developed by reviewing a crosswalk of the existing goals of CBI, the program level educational competencies, and the first level of the Accreditation Council for Graduate Medical Education (ACGME) residency milestones related to critical thinking skills of four specialties, Medicine, Family & Community Medicine, Pediatrics, and Obstetrics and Gynecology.

The crosswalk process enabled the team to identify similar expectations between the original CBI modules, the goals of the undergraduate medical education program and the expectations for performance in residency. Once the course goals were created, the team then identified the ‘acceptable evidence’ associated with the course goals by asking the questions ‘How will I know if students have achieved the desired results?’ and ‘What will I accept as evidence of student understanding and proficiency?’ The discussion of desired results and evidence for those results prompted the course team to discuss the possibility of improving the connection between the formative feedback and assessment in the CRC to the ACGME Milestones approach used in residency programs. The course team also relied significantly on work implemented at the University of New Mexico [6].

The ACGME identified six core competencies for medical education: practice-based learning and improvement, patient care and procedural skills, system-based practice, medical knowledge, interpersonal and communication skills, and professionalism [7]. Because CRC provided the best opportunity in our pre-clinical curriculum for faculty to observe the performance of individual students and provide feedback, CRC was used to assess the competencies of Medical Knowledge (in this case, clinical reasoning), Interpersonal and Communication Skills, Practice-based Learning and Improvement, and Professionalism.

The ACGME residency milestones were implemented in 2013. The milestones created a developmental framework for the assessment of professional skill development in residency training and provided more meaningful assessment and outcome measures. Using these milestones as a reference, the CRC team aimed to create a student performance assessment rubric that was developmental, clearly described the trajectory of performance, skills and behaviors we expected students to develop throughout the three semesters of the course, and included the future target for performance (connecting each behavior to related expectations in clerkship).

The course team focused on behaviors we expected students to be able to demonstrate, identifying each as a ‘desired result’ element and aligning these with four levels of performance: pre-emergent, emerging, acquiring, and mastering. Each level was defined by a descriptive anchor. Students received a mid-semester and an end-semester assessment (weighted 20% and 80% toward their final grade). As the students proceeded through the three semesters of the course, the threshold assessment rating necessary to pass the course increased.

The first iteration of the assessment rubric included 12 items in four competencies (Table 1). The full version of the assessment rubric with the descriptive anchors is provided in Appendix 1.

**Table 1.** First version of assessment rubric items.

**Medical Knowledge**
Identifies the pertinent facts of a clinical case (MK 1)
Collects and records information about a clinical case in a manner that supports the development of a differential diagnosis (MK 2)
Develops multiple working Hypotheses (i.e., differential diagnosis) related to clinical diagnosis (MK 3)
**Interpersonal and Communication Skills**
Provides a rationale for each hypothesis (ICS4)
Provides constructive feedback to peers (ICS 5)
Participates in the problem solving process (ICS 6)
**Problem-Based Learning and Improvement**
Asks relevant questions about the case in order to identify gaps in knowledge necessary to resolve the problem (PBLI 7)
Identifies and cites appropriate sources of research (PBLI 8)
Reflects on case and process, including identifying cognitive errors when they arise (PBLI 9)
Demonstrates awareness or insight into own weakness and limitations (PBLI 10)
**Professionalism**
Acknowledges differences of opinion and perspective among group members (PRO 11)
Appropriately documents work; research; or contributions to the group process (PRO 12)
*0 = Pre-Emergent, 1-Emerging, 2 = Acquiring, 3 = Mastery*

Once the draft rubric was created the course team implemented an initial review process. The course team provided five faculty facilitators with written examples of student reasoning that demonstrated different performance levels. The team used multiple student case entries within ThinkShare from three students over a year of CBI. All case entries were from students who took the course in prior years and were blinded to the reviewer. The team used qualitative analysis to identify themes in post-case reflections that demonstrated students’ engagement in a range of analytical, evaluative and other forms of higher order thinking and metacognitive engagement as defined by Bloom’s Taxonomy Revised and Shraw and Dennison [8]. Codes were derived to define each theme. Using Atlas.ti, we analyzed entries from one year of students and then selected eight post-case reflections for each of two cases that demonstrated the three levels of performance described in the new pilot rubric (emerging, acquiring, and mastering).

The CRC team asked the faculty reviewers to assess these student entries using the new assessment rubric. The faculty scored the entries, and provided feedback on timing, ease of use, and appropriateness. Their feedback was used by the CRC team to make adjustments to the rubric. The CRC team reviewed the assessment ratings between each reviewer and relative to the prior assessment of performance (which was blinded to the pilot faculty reviewers) and subsequently revised the assessment rubric based on faculty reviewer feedback.

The assessment rubric was used for the first time in the fall of 2015 when the course was implemented in its longitudinal format. Faculty facilitators for the CRC course were trained to use the assessment rubric by the Director of Faculty Development and the Manager for Student Assessment, including applying the rubric to the sample student entries from the pilot. The faculty facilitators for each student group assessed student performance at mid- and end of the semester using the new assessment rubric.

In addition to reviewing the descriptive data, the course team continued the iterative improvement process that began during the first year of implementation to gather faculty facilitator feedback beginning with a focus group. Based on feedback from the focus group, the CRC team members paired with two faculty facilitators to make additional revisions to the items and behavioral anchors in each competency area. This process led to a second version of the assessment rubric with nine items in four competencies (Appendix 2). This version of the rubric was then used for the assessment of students at every mid-semester and end of semester evaluation from that point forward.

Ethical approval was waived by the University of Arizona Institutional Review Board.

## Results

After the first semester implementation of the assessment rubric, the CRC team identified a key question to evaluate the rubric: did the assessment rubric distinguish between different levels of student performance in each competency? In order to answer this question, the team implemented a systematic approach based on a descriptive analysis of the mid- and end of semester assessment ratings of student performance. Since the assessment was implemented with first-year students in their first semester of medical school, the team expected that ratings would increase from the mid- to the end of the semester. Tables 2 and 3 present our descriptive data from the mid-and end of semester assessments for the 116 students enrolled in the course. The CRC team reviewed the descriptive data and interpreted the results as promising. At mid-semester assessment, the distribution of the assessment ratings was evenly split between 1 = emerging and 2 = acquiring for most individual items with very few ratings of 0 = pre-emergent or 3 = Mastery. At the end of the semester, this distribution shifted towards more students receiving ratings of 2 = acquiring for most of the items. This is reflected in the change in median scores for each competency. Table 3 presents the results of a matched analysis using a Wilcoxon-signed rank test. The CRC team used this data to understand the trend for individual students matched scores. This data shows that from mid- to end-of-semester, over half the students (ranging from 53% to 62%) received a higher competency score at the end-of-the semester.

**Table 2.** Descriptive statistics mid- and end-semester assessment.

	Mid-Semester (N = 116)				End-Semester (N = 116)			
	Mean	Median	Mode	SD	Mean	Median	Mode	SD
Medical Knowledge	1.56	1.67	1.0	0.5	1.83	2.0	2.0	0.55
Interpersonal and Communication Skills	1.48	1.33	1.33	0.44	1.82	2.0	2.0	0.53
Problem-Based Learning and Improvement	1.52	1.50	1.00	0.52	1.84	2.0	2.0	0.57
Professionalism	1.65	1.50	2.0	0.56	2.02	2.0	2.0	0.65

*0 = Pre-Emergent, 1 = Emergent, 2 = Acquiring, 3 = Mastering*

**Table 3.** Number of students with decrease, tied, or increased total rating score by competency.

	(N = 116)		
	Rating Decreased	Rating Stayed the Same	Rating Increased
Medical Knowledge	19 (16.4%)	35 (30%)	62 (53.4%)
Interpersonal and Communication Skills	14 (12.1%)	32 (27.6%)	70 (60.3%)
Problem-Based Learning and Improvement	21 (18.1%)	23 (19.8%)	72 (62.1%)
Professionalism	25 (21.5%)	29 (25%)	62 (53.4%)

*0 = Pre-Emergent, 1 = Emergent, 2 = Acquiring, 3 = Mastering*

## Discussion

The CRC assessment rubric has undergone revisions over the first three years of the course based on feedback from students and faculty. Continued work to describe the developmental trajectory of clinical reasoning skills for pre-clinical medical students is necessary and will inform future iterations of this assessment. We believe that this rubric has allowed students who are in the early stages of their clinical reasoning development to visualize their trajectory and has provided faculty facilitators a framework from which to provide meaningful and concrete feedback. The expected development of these skills in early learners is well described by our assessment rubric.

CRC continues to be an integral component of our medical school curriculum though the leadership of the course has changed. The new course directors have continued to build on the developmental approach integrated in the rubric. We do recognize the need for further testing on the validity and reliability of our rubric. Structural and organizational limitations in managing a small group course, facilitated by faculty, for an entire cohort of students presented a logistical challenge to implementing additional reliability testing. The course did implement an online Health Sciences Reasoning Test (HSRT) with the first cohort of students with the plan to analyze the possible relationship between the rubric and student results on the HSRT. However, this analysis was not able to be conducted due to organizational constraints. These limitations highlight the importance of dedicating enough time and focus to the follow up reliability and validity analysis of any new tool [9].

Another limitation relates to our findings of the mid- and end of semester ratings. It is possible that the differences in ratings over time could be due to several factors including changes in rater expectations or differences in rater training. In addition, as the weighting of mid-semester assessment was worth 20% of their final grade while the end of semester was worth 80%, students may have put forth more effort into their work at the end of the semester.

Future work and applications for our clinical reasoning rubric could be focused on use during clerkship rotations. We did include ‘Clerkship Level’ goals for each behavior which could be used to modify the rubric with higher level expectations for learners on their clinical rotations.

The course design process and the development and pilot process for the assessment rubric would not have been possible without the commitment of the multi-disciplinary CRC team members. Our experience emphasizes the need for Colleges of Medicine to provide structural support to the course design and implementation process. This includes valuing the time and effort dedicated to course design by individuals from multiple disciplines with advanced degrees in the basic sciences, clinical medicine, education and public health and with expertise in instructional design, assessment, and course/curriculum evaluation.

## Appendix Appendix 1

### Milestones of Observable Behaviors for Clinical Reasoning Course

**Table:**

Milestones (points per behavior)			
Pre-Emergent	Emerging (1 point)	Acquiring (2 points)	Mastering (3 points)
**1. Identifies the pertinent facts of a clinical case (MK).**			
Does not note case information	Repeats Initial History (IH) with little or no editing. OR Restates IH, but omits important information. Does not identify pertinent clinical facts (i.e., no mention of pertinent positives/negatives, risk factors, social/cultural factors, etc.)	Restates IH in a way that captures pertinent clinical facts. Begins to distinguish normal from abnormal findings. Begins to identify relevant negative findings.	Defines problem by identifying pertinent positives and negatives, risk factors, etc. from Initial History (IH). Clearly distinguishes normal from abnormal findings. Omits irrelevant information. Explicitly identifies emergent concerns/possible emergencies.
*Comments*:			
**2. Collects and records information about a clinical case in a manner that supports the development of a differential diagnosis (MK).**			
Does not attempt to collect additional information about case. Additional information is incomplete and is presented with no organization.	Collects data, but not sufficient to explain case. Requests little or no additional information, or gives no rationale for request. Information presented is not well organized.	Usually collects data in an organized manner, but sometimes uses unfocused ‘data grab’ in seeking additional information (‘I would get a complete medical, family, social, and medication history’) or seeks additional information with limited rationale. Organizes most of the case information using a clearly apparent system, such as the SOAP format.	Demonstrates focus and efficiency when collecting data by seeking that additional information that can distinguish among his/her different hypotheses. All case information is well organized (e.g., follows SOAP format) and supports development of a differential diagnosis.
*Comments*:			
**3. Develops multiple working hypotheses (i.e., a differential diagnosis) related to clinical diagnosis (MK).**			
Proposes a single or very few hypotheses.	Does not develop enough hypotheses to progress through the case. Perseverates on hypotheses despite contradictory evidence.	Develops multiple working hypotheses regarding a clinical diagnosis.	Develops multiple working hypotheses regarding a clinical diagnosis in a manner demonstrates an organized approach or structure (e.g ranks or groups hypotheses by likelihood, risk level, etc.).
*Comments*:			
**4. Provides a rationale for each hypothesis (IPS).**			
Provides no rationales for most or all hypotheses.	Provides insufficient rationales for hypotheses. Uses opinion or unsupported hunches (faith-based problem solving: ‘I believe…’).	Usually articulates reasoning by providing a relevant basic science rationale/explanation for each hypothesis. Usually relates key elements of the case to DDX.	Consistently provides a relevant basic science rationale/explanation for each hypothesis. Includes at least an initial assessment of likelihood of each hypothesis for this case based on available case information. Identifies and tolerates low-probability hypotheses with rationale. Includes an explicit statement about how well each hypothesis fits this patient. Identifies case information that doesn’t fit a given hypothesis.
*Comments*:			
**5. Provides constructive feedback to peers (IPS).**			
Provides no feedback to peers.	Provides feedback to peers occasionally. Feedback provided to peers is insufficient or not constructive (e.g., ‘Nice job.’).	Routinely provides constructive feedback to all group members.	Provides constructive feedback for individual group members and offers constructive feedback on the group’s functioning, including strategies for improvement.
*Comments*:			
**6. Participates in group problem-solving process (IPS).**			
Does not contribute to group discussions.	ThinkShare entries address just the basic elements of the case, with limited explanation of thinking. Participates in group discussions occasionally, but not regularly.	ThinkShare entries demonstrate sustained effort to understand most aspects of case. Usually participates in the group problem solving process.	ThinkShare are exemplary: clear, thorough, organized, and thoughtful. Helps to lead the group discussion without dominating.
*Comments*:			
**7. Asks relevant questions about the case in order to identify gaps in knowledge necessary to resolve the problem (PLI).**			
Seldom or never asks relevant questions or identifies gaps in knowledge necessary to resolve the problem.	Occasionally asks relevant questions or identifies gaps in knowledge necessary to resolve the problem. Relies on information from group members or assistance from facilitator in order to formulate questions or identify requisite knowledge.	Usually asks relevant questions about the case. Is able to identify gaps in knowledge necessary to advance the case.	Consistently asks relevant questions about the case. Routinely identifies gaps in knowledge necessary to advance the case. Uses identified gaps in knowledge to organize data collection.
*Comments*:			
**8. Identifies and cites appropriate sources of research (PLI).**			
Does not use or does not cite outside sources of information.	Cites few sources. Uses weak or inappropriate sources. Sometimes does not cite sources.	Cites source(s) used but does not comment on credibility.	Uses and cites appropriate sources and comments on their value.
*Comments*:			
**9. Reflects on case and process, including identifying cognitive errors when they arise (PLI).**			
Provides superficial or dismissive comments in reflections.	Provides brief or otherwise limited comments in reflection (e.g., ‘This case taught me to think more clearly.’). Focuses almost entirely on content of case and content knowledge acquired. Seldom recognizes own or others’ cognitive error (e.g., premature closure).	Comments on what s/he did well or poorly in working on the case. Comments on what aspects of the case made it challenging or easier. Sometimes includes a plan for future improvement. Begins to recognize and address own and others’ cognitive errors.	Describes the approach s/he used in this case, and comments on relative strengths of chosen approach compared with others. Identifies strategies s/he used to make progress/get un-stuck. Outlines specific plan for improving in future cases (not just, ‘Next time I’m going to do better.’). Consistently recognizes and addresses own and others’ cognitive errors.
*Comments*:			
**10. Demonstrates awareness or insight into own weaknesses and limitations, and seeks help to address them (PLI).**			
Demonstrates no awareness of own weaknesses or limitations.	Occasionally demonstrates awareness or insight into own weaknesses and limitations. Only seeks help to address weaknesses when prompted by others.	Regularly demonstrates awareness of own weaknesses and limitations. Sometimes seeks help to address weaknesses/limitations.	Consistently demonstrates awareness of own weaknesses and limitations. Consistently seeks help when needed. Shows dedication to improvement in self and others.
*Comments*:			
**11. Acknowledges differences of opinion and perspective among group members (PRO).**			
Demonstrates belligerence toward or belittles those with different opinions or perspectives.	Acknowledges differences of opinion and perspective among group members, but with some difficulty.	Respectfully acknowledges differences of opinion, perspective, and capabilities among group members.	Models respectful behaviors for others and actively coaches group members.
*Comments*:			
**12. Appropriately documents work; research; or contributions to the group process (PRO).**			
Seldom or never documents work, research, or contributions to the group process.	Occasionally documents work, research, or contributions to the group process.	Regularly documents work, research, or contributions to the group process, although with some lapses or oversights.	Consistently and appropriately documents own and others’ work and contributions, accurately cites research, and recognizes others’ contributions to own work and thinking.
*Comments*:

**Table:**

**1. Identifies, collects, and utilizes the pertinent information of a clinical case (MK)**. **This item focuses on the student organizing his/her information and thinking.**			
Pre-Emergent	Emerging (1 point)	Acquiring (2 points)	Mastering (3 points)
*Has not achieved the Emerging level*. Restates case information with little or no interpretation. Requests little or no additional information, or gives no rationale for request, ‘data grab.’ Omits pertinent information.	Restates case information in a way that captures pertinent clinical facts. Requests additional data with some rationale.	Begins to use semantic qualifiers to present case information. Distinguishes normal from abnormal findings. Identifies relevant negative findings. Identifies emergent conditions and concerns. Requests pertinent data with clear and logical rationale.	Defines problem clearly by using appropriate semantic qualifiers and identifying pertinent positives and negatives. Organizes data and identifies key finding or constellation of findings. Omits irrelevant information. Requests for data are comprehensive, essential, and prioritized by context. Rationales are explained clearly and succinctly.

*CLERKSHIP LEVEL (for reference; not expected of pre-clerkship students): Collects important data, including pertinent positive and negatives, in a systematic and efficient manner. Analyzes this data, synthesizes it into a focused problem list, and identifies key finding or constellation of findings. Presents this information in a clear, concise, organized format.*

**Table:**

**2. Develops and prioritizes hypotheses related to clinical diagnosis (MK).**			
Pre-Emergent	Emerging (1 point)	Acquiring (2 points)	Mastering (3 points)
*Has not achieved the Emerging level*. Hypotheses are not grounded in reasonable rationales. Perseverates on hypotheses despite contradictory evidence.	Lists at least 3 hypotheses with relevant rationales, even if rationales are basic.	Develops multiple working hypotheses, articulates reasoning using relevant basic science/explanations. Reconsiders differential diagnoses after receiving new case information, integrates new key features into reasoning.	Prioritizes multiple working hypotheses and articulates clear rationale for the rank ordering of the hypotheses. Includes an explicit statement about how well each hypothesis fits this patient and discusses reasoning of low likelihood hypotheses. Identifies case information that doesn’t fit a given hypothesis. Develops diagnostic strategy to rule in or rule out hypotheses.

*CLERKSHIP LEVEL (for reference; not expected of pre-clerkship students): Uses key features and problem list to develop prioritized, realistically plausible differential diagnoses using a systematic approach. Uses new information and data to reprioritize, reconsider, and develop new hypotheses. Considers ‘must not miss’ diagnoses as well as most common or likely diagnoses. Uses basic science principles, knowledge of likelihood of diagnoses, and test characteristics to develop organized diagnostic strategy to confirm or dismiss differential diagnoses.*

## Appendix Appendix 2

### Milestones of Observable Behaviors for Clinical Reasoning Course

**Table:**

**3. Provides constructive feedback to peers (IPS)**.			
Pre-Emergent	Emerging (1 point)	Acquiring (2 points)	Mastering (3 points)
*Has not achieved the Emerging level*. Does not provide feedback to peers in group sessions or through ThinkShare entries or comments.	Comments on peer entries in ThinkShare or peer ideas during group sessions, but does not go beyond evaluative remarks, such as ‘nice job.’	Provides constructive comments regarding group ideas or process in ThinkShare or during group sessions. Comments address the group process or functioning by describing specific, observable behaviors, approaches, or dynamics.	Provides constructive feedback to individual peers in ThinkShare or during group sessions, such as explaining what they thought their peer did well or identifying possible errors, posing questions about peer approaches to problem-solving, contributions to group, etc. Feedback includes strategies or suggestions for improvement.

*CLERKSHIP LEVEL (for reference; not expected of pre-clerkship students): Provides actionable feedback to peers that contributes to learning and functioning of team.*

**Table:**

**4. Participates in group problem-solving process (IPS).**			
Pre-Emergent	Emerging (1 point)	Acquiring (2 points)	Mastering (3 points)
*Has not achieved the Emerging level*. Does not provide input during group sessions. ThinkShare entries do not reflect consideration of peer ideas or approaches.	ThinkShare entries reference peer ideas or approaches. Participates in group problem-solving process (e.g., offers ideas or explanations, serves as a scribe, suggests resources).	ThinkShare entries reference how the student used peer ideas to aid his/her approach to cases. Or student suggests constructive ideas that promote or redirect group discussion, evaluates resources, or begins to take the lead in group discussions.	ThinkShare entries or contributions during group sessions model problem-solving in a clear, thorough, organized, and thoughtful manner. Or during group sessions, consistently suggests ideas that help lead or redirect group discussion without dominating.

*CLERKSHIP LEVEL (for reference; not expected of pre-clerkship students): Participates in problem-solving as a member of the clinical team. Presents ideas in a thoughtful, organized, clear manner.*

**Table:**

**5. Asks relevant questions and identifies gaps in knowledge needed to solve the case (PLI). *This item is intended to focus on the student identifying and filling gaps in his/her medical knowledge. The scale is based on the frequency of performing the behavior***.			
Pre-Emergent	Emerging (1 point)	Acquiring (2 points)	Mastering (3 points)
*Has not achieved the Emerging level.*	Occasionally asks relevant questions or identifies gaps in knowledge necessary to solve the case.	Usually asks relevant questions about the case and identifies gaps in knowledge.	Almost always asks relevant questions and identifies gaps in knowledge needed to solve the case.

*CLERKSHIP LEVEL (for reference; not expected of pre-clerkship students): Routinely identifies gaps in medical knowledge and performs self-directed research to find the information. Evaluates new information in context of clinical problem and utilizes information to develop plan of care and treatment. Incorporates new information into knowledge base and uses this when approaching future similar problems.*

**Table:**

**6. Uses and properly cites high-quality sources for research (PLI). *This scale is intended to combine the frequency and accuracy of citing sources and the strength of the sources used.***			
Pre-Emergent	Emerging (1 point)	Acquiring (2 points)	Mastering (3 points)
*Has not achieved the Emerging level.*	Sometimes provides proper citation of sources. Sometimes uses sources commonly accepted in academic medicine.	Usually provides proper citation of sources. Usually uses sources commonly accepted in academic medicine.	Almost always provides proper citation of sources. Almost always uses sources commonly accepted in academic medicine.

*CLERKSHIP LEVEL (for reference; not expected of pre-clerkship students): Uses multiple sources for research and is able to perform focused and efficient search for information. Can evaluate sources regarding strength of study, application to current clinical problem, etc.*

**Table:**

**7. Reflects on his/her reasoning process and strategizes to improve it (PLI). *This scale is based on both the frequency of one aspect of the behavior and the acquisition of a new, second skill for the behavior. It is intended to focus on the student’s comments or reflections on his/her reasoning process and not comments on case content or the student’s medical knowledge.***			
Pre-Emergent	Emerging (1 point)	Acquiring (2 points)	Mastering (3 points)
*Has not achieved the Emerging level.*	Sometimes comments on strengths/weaknesses of his/her reasoning or approach to case.	Usually comments on strengths/weaknesses of his/her reasoning or approach to case. Sometimes outlines a specific strategy for improving his/her reasoning process and evaluates its effectiveness.	Almost always comments on strengths/weaknesses of his/her reasoning or approach to case. Often outlines a specific strategy for improving his/her reasoning process and evaluates its effectiveness.

*CLERKSHIP LEVEL (for reference; not expected of pre-clerkship students): Routinely performs self-directed reflection on academic and clinical performance. Identifies strengths and weaknesses and develops strategy to improve performance. Implements strategy in future situations and reflects on effectiveness of this strategy.*

**Table:**

**8. Acts upon feedback from facilitator and peers (PRO). *This scale is based on how quickly and how often the student responds to explicit or implicit feedback. Note: Students are required to participate in the scheduled formative-feedback meetings with facilitators. Failure to attend those sessions would be an attendance issue and should not be included in this assessment survey. This item focuses on the student’s response to feedback.***			
Pre-Emergent	Emerging (1 point)	Acquiring (2 points)	Mastering (3 points)
*Has not achieved the Emerging level.*	Promptly acts upon explicit feedback or input from facilitator.	Sometimes or eventually acts upon implied or non-verbal input/feedback from peers or facilitator.	Usually acts promptly upon implied or non-verbal input/feedback from peers or facilitator.

*CLERKSHIP LEVEL (for reference; not expected of pre-clerkship students): Routinely seeks feedback on performance and incorporates feedback into strategies for improved performance or continued strong performance. Maintains situational and social awareness to perceive non-verbal or implied feedback from peers, attending physicians, and patients and acts upon feedback accordingly to perform as an effective member of the clinical team. Provides feedback to peers and patients in professional manner.*

**Table:**

**9. Acknowledges differences of opinion and perspective among group members (PRO). *This scale is based on the quality of performance.***			
Pre-Emergent	Emerging (1 point)	Acquiring (2 points)	Mastering (3 points)
*Has not achieved the Emerging level.*	Acknowledges differences of opinion or perspective among group members, but with some difficulty.	Articulates differences of opinion or perspective among group members.	Models respectful behaviors for others or actively assists group in reconciling differences of opinion or perspective.
*CLERKSHIP LEVEL (for reference; not expected of pre-clerkship students): Always conducts himself/herself in manner that respects all members of clinical team and patients.*			

**☐ YES ☐ NO This student submitted ThinkShare entries for all steps of all cases.**

**☐ YES ☐ NO This student met with the facilitator for individual, in-person formative feedback.**

***Please provide written comments about this student’s performance during this assessment period*:**
